# Historical trend on seed amino acid concentration does not follow protein changes in soybeans

**DOI:** 10.1038/s41598-020-74734-1

**Published:** 2020-10-19

**Authors:** Andre Froes de Borja Reis, Santiago Tamagno, Luiz H. Moro Rosso, Osler A. Ortez, Seth Naeve, Ignacio A. Ciampitti

**Affiliations:** 1 Department of Agronomy, Kansas State University, Manhattan, KS USA; 2 Department of Agronomy and Horticulture, University of Nebraska, Lincoln, NE USA; 3 Department of Agronomy and Plant Genetics, University of Minnesota, Saint Paul, MN USA; 4 Present Address: Department of Plant Sciences, University of California, Davis, CA USA

**Keywords:** Plant sciences, Plant physiology

## Abstract

Soybean [*Glycine max* (L.) Merr.] is the most important oilseed crop for animal industry due to its high protein concentration and high relative abundance of essential and non-essential amino acids (AAs). However, the selection for high-yielding genotypes has reduced seed protein concentration over time, and little is known about its impact on AAs. The aim of this research was to determine the genetic shifts of seed composition for 18 AAs in 13 soybean genotypes released between 1980 and 2014. Additionally, we tested the effect of nitrogen (N) fertilization on protein and AAs trends. Soybean genotypes were grown in field conditions during two seasons under a control (0 N) and a N-fertilized treatment receiving 670 kg N ha^−1^. Seed yield increased 50% and protein decreased 1.2% comparing the oldest and newest genotypes. The application of N fertilizer did not significantly affect protein and AAs concentrations. Leucine, proline, cysteine, and tryptophan concentrations were not influenced by genotype. The other AAs concentrations showed linear rates of decrease over time ranging from − 0.021 to − 0.001 g kg^−1^ year^−1^. The shifts of 11 AAs (some essentials such as lysine, tryptophan, and threonine) displayed a relative-to-protein increasing concentration. These results provide a quantitative assessment of the trade-off between yield improvement and seed AAs concentrations and will enable future genetic yield gain without overlooking seed nutritional value.

## Introduction

Soybean [*Glycine max* (L.) Merr.] is a major oilseed and protein crop which is responsible for roughly 70% of the world’s plant-based meal^1^. Soybean meal is the by-product of oil extraction and provides a high-quality protein for animal feed and other uses^2^. The quality of the protein is defined by the relative constitution of amino acids (AAs) and the profile of essential and non-essential AAs^3,4^. From a production viewpoint, improvements in agronomic management and breeding have led to increases in soybean yields^5^ in parallel with the global demand for this crop. A main challenge in soybean breeding has been to improve yields while maintaining market standard for protein concentration (e.g., soybean meal levels) despite the consistent trade-off between seed yield and protein concentration^6,7^.

Average protein concentration in current commercially available soybean genotypes ranges between 33 and 39% (on 130 g kg^−1^ moisture content)^8^. Commonly, 18 AAs (essential and non-essential) are reported as soybeans protein constituents^9^. Asparagine and glutamine are the most abundant, corresponding to approximately 12% and 18%, respectively, of total AAs; however, they are considered non-essential for humans and monogastric animals^10^. The essential AAs constitute a relatively smaller proportion of seeds including lysine, threonine, tryptophan, isoleucine, leucine, histidine, phenylalanine, valine, and the sulfur amino acids cysteine and methionine^11,12^.

The relationship between seed protein and AA concentration varies according to each AA. For instance, lysine, methionine, cysteine, tryptophan, and threonine are negatively correlated with seed protein concentration, whereas arginine and glutamic acid increase with seed protein concentration^9,13^. In China, significant negative trends for glutamic acid, histidine, and arginine were reported in soybean genotypes released between 1923 and 2007^14^. However, the study did not contemplate the effect of N on seed composition and the changes of AAs relative to the modifications in seed protein concentration.

At the plant level, soybean seed composition is the result of complex genotype and environment interactions^15,16^. During the seed-filling period, protein synthesis occurs in the developing seeds on the basis of carbon and N compounds provided by the mother plant. Seed N demand represents about 75% of the total plant N uptake during the season^17^, being mostly remobilized from vegetative organs^18^ and concurrently assimilated from soil mineral N and biological N fixation. Hence, N accumulated prior to seed formation will be a predominant source for protein synthesis in seeds^19^. The effect of N fertilization on seed protein concentration has been studied, but response seems to be erratic^16^ and historical trends in protein dilution could not be reversed even in heavily N-fertilized environments^20^. However, the degree to which individual AAs respond to N application across historical genotypes remains unknown.

Therefore, considering a historical set of soybean genotypes released between 1980 and 2014 in the United States, the aims of this research were as follows: (i) determine the genetic gain of 18 AAs concentrations in soybean seeds, (ii) evaluate the response in seed AA profile to N fertilization, and (iii) compare the AAs genetic gain by clustering those AAs presenting similar shifts (in both absolute and relative terms) relative to seed weight and protein. This information contributes to our ability to understand the magnitude and potential determinants of seed composition changes, therefore enabling future investigations for a more effective selection of seed nutritional value in soybeans.

## Results

### Seed yield and protein genetic gain in historical genotypes

**Figure 1:**
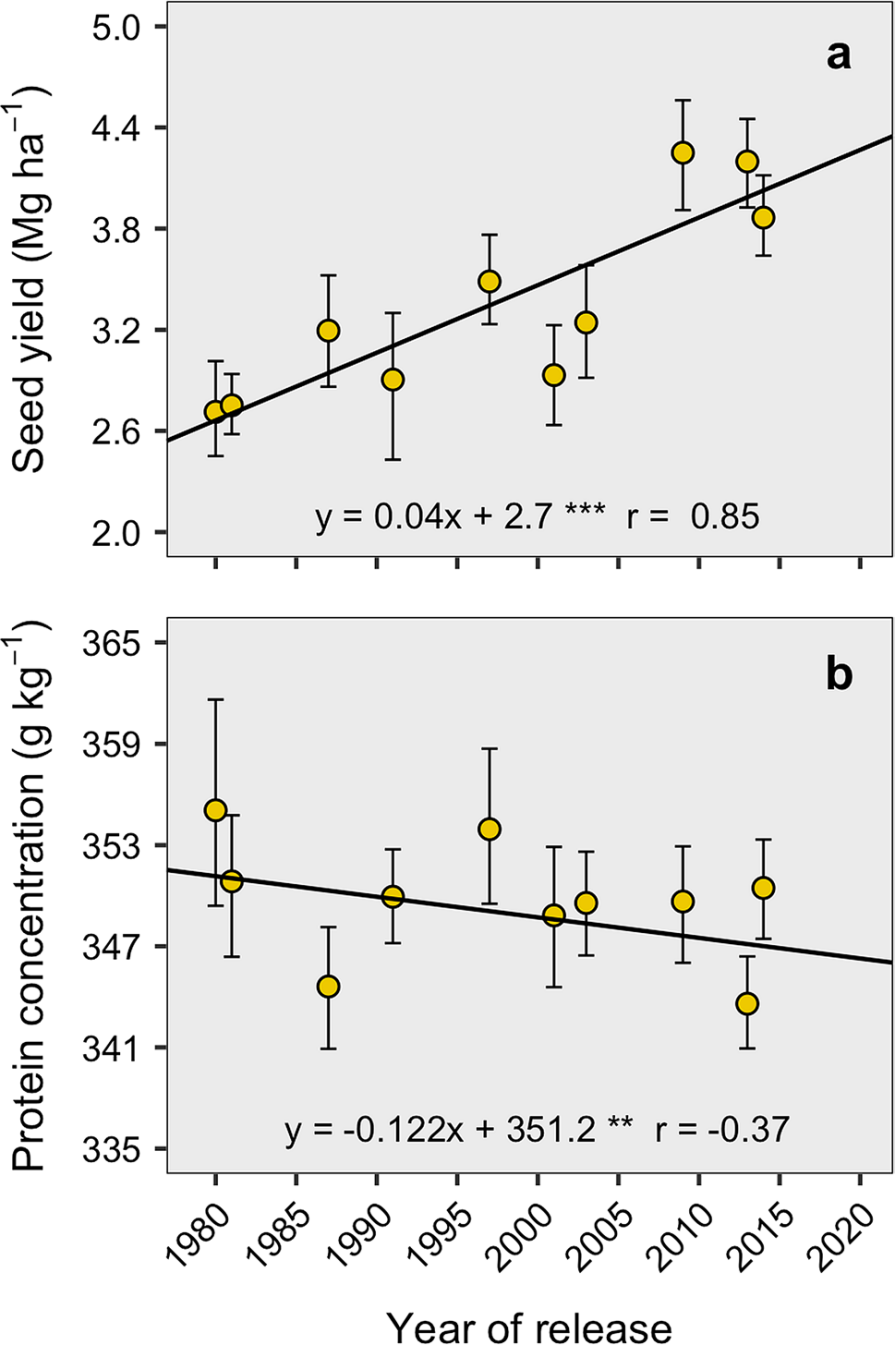
Relationship between seed yield (13 g kg^*−*1^ of moisture content) (**a**), protein concentration (**b**), and year of release of 13 genotypes released from 1980 to 2014 period. Each point represents the mean of both N treatments and genotypes (if more than one per year). Solid black lines denote the best fitted linear model. Asterisks indicate significance of the coefficient: ***P < 0.001; **P < 0.01. The r value represents the Pearson correlation.

The absolute genetic gain was given by the relationship between the crop trait and the genotype year of release. The mean crop yield genetic gain was 0.04 Mg ha^−1^ year^−1^ (Fig. 1a) regardless of the N fertilization treatment (Table 1). Seed yield ranged between 2.7 and 4.1 Mg ha^−1^ with an estimated yield increase of approximately 50% from 1980 to 2014. Protein absolute genetic gain decreased at a rate of 0.122 g kg^−1^ year^−1^ (Fig. 1b). Similar to yield, seed protein concentration was not affected either by the N fertilization nor its interaction with year of release (Table 1). The average protein concentration was 349 g kg^−1^, with an overall reduction of 1.18% when considering the entire evaluation period, and a total decrease of − 4.15 g kg^−1^ (Fig. 1b).

**Table 1:** Analysis of variance *F*-test probabilities for nitrogen fertilization and genotype year of release affecting soybean traits.

Crop Trait	N fertilization (N)	Genotype year of release (YR)	N x YR
Yield	0.33^ns^	< 0.001***	0.31^ns^
Protein	0.26^ns^	< 0.01**	0.83^ns^
Glutamic acid	0.39^ns^	< 0.01**	0.66^ns^
Aspartic acid	0.28^ns^	< 0.01**	0.72^ns^
Leucine	0.11^ns^	0.03*	0.87^ns^
Arginine	0.40^ns^	< 0.01*	0.67^ns^
Lysine	0.22^ns^	< 0.01**	0.86^ns^
Phenylalanine	0.13^ns^	< 0.01*	0.78^ns^
Proline	0.12^ns^	0.11^ns^	0.95^ns^
Isoleucine	0.24^ns^	0.02*	0.99^ns^
Valine	0.24^ns^	0.02*	0.98^ns^
Serine	0.27^ns^	< 0.01**	0.97^ns^
Glycine	0.15^ns^	0.01*	0.72^ns^
Alanine	0.12^ns^	0.02*	0.84^ns^
Threonine	0.13^ns^	0.02*	0.86^ns^
Tyrosine	0.27^ns^	0.02*	0.65^ns^
Histidine	0.17^ns^	< 0.01**	0.98^ns^
Cysteine	0.53^ns^	0.17^ns^	0.88^ns^
Methionine	0.46^ns^	0.02*	0.52^ns^
Tryptophan	0.70^ns^	0.24^ns^	0.35^ns^

### Amino acid genetic gain over time

**Figure 2:**
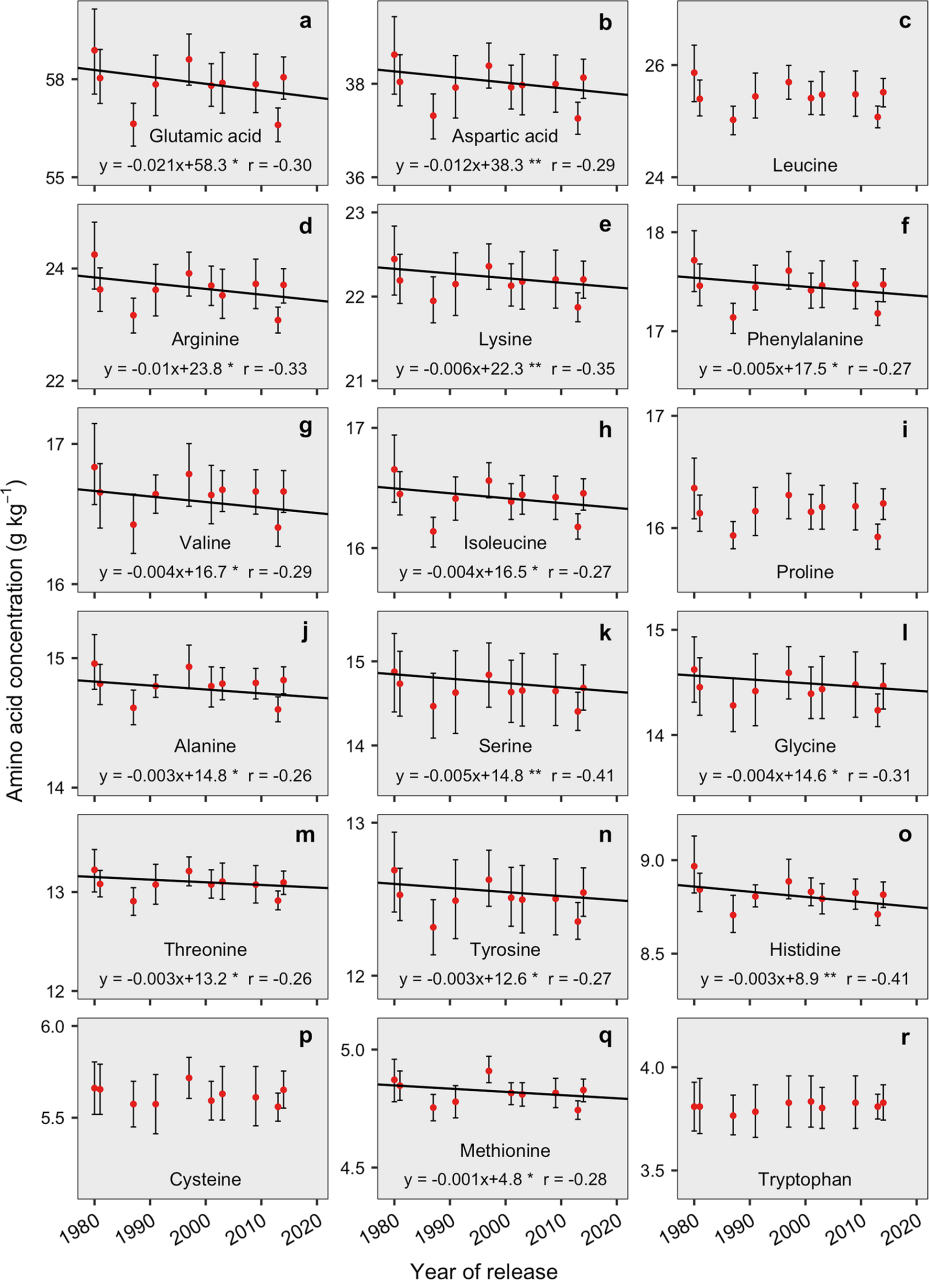
Relationship between glutamic acid (**a**), aspartic acid (**b**), leucine (**c**), arginine (**d**), lysine (**e**), phenylalanine (**f**), valine (**g**), isoleucine (**h**), proline (**i**), alanine (**j**), serine (**k**), glycine (**l**), threonine (**m**), tyrosine (**n**), histidine (**o**), cysteine (**p**), methionine (**q**), tryptophan (**r**), and year of release of 13 soybean genotypes released from 1980 to 2014. Each point represents the average between the two N treatments and genotypes (if more than one per year). Solid black lines denote the AA genetic gain fitted model. Asterisks indicate significance of the coefficient: ***P < 0.001; **P < 0.01; *P < 0.05. *The* r *value* represents the Pearson correlation*.*

The majority of the AAs presented a significant decrease in absolute genetic gain (Table 1, Fig. 2), with the exception of leucine, proline, cysteine, and tryptophan (Fig. 2c, i, p, r). Similarly to yield and protein results, the N fertilization did not affect the AA trends over time. Glutamic acid displayed a rate of − 0.021 g kg^−1^ year^−1^, with a decrease of 1.22% over the 1980 to 2014 timeframe (Fig. 2a). The concentration of aspartic acid decreased by 1.07% at a rate of − 0.012 g kg^−1^ year^−1^ (Fig. 2b). The other non-essential AAs (arginine, alanine, serine, glycine, and tyrosine) followed the same overall decreasing trend. Arginine concentration decreased by 1.43% in the selected time period, with a rate of − 0.01 g kg^−1^ year^−1^ (Fig. 2d), whereas alanine concentration decreased 0.8% with a rate of − 0.003 g kg^−1^ year^−1^ (Fig. 2j). Serine and glycine concentrations decreased at a rate of − 0.005 g kg^−1^ year^−1^ and 0.004 g kg^−1^ year^−1^, respectively, (Fig. 2k, l) with an overall reduction of 1.21% and 0.88%, respectively, over the evaluated period of time.

In the essential AAs group, lysine concentration decreased by 0.92% for the evaluated period at a rate of – 0.006 g kg^−1^ year^−1^ (Fig. 2e). Phenylalanine decreased at a rate of − 0.005 g kg^−1^ year^−1^ with concentration ranging from 17.54 to 17.37 g kg^−1^ (Fig. 2f). Valine decreased by 0.81% with a rate of − 0.004 g kg^−1^ year^−1^ (Fig. 2g). Isoleucine, threonine, and histidine showed rates of − 0.004, − 0.003, and − 0.003 g kg^−1^ year^−1^ with an average decrease of 0.85%, 1.03%, and 1.15%, respectively (Fig. 2h, m, o). Both leucine and tryptophan concentrations remained steady across genotypes with averages of 25.5 and 3.8 g kg^−1^, respectively (Fig. 2c, r). For the sulfur amino acids, only methionine concentration was influenced by genotype’s year of release. Genetic gain for methionine was − 0.001 g kg^−1^ year^−1^ in seed concentration, with the modern genotype attaining 0.71% less methionine than the oldest genotype evaluated in this study (Fig. 2q). Cysteine was constant in soybean seeds across the years of release with a mean concentration of 5.6 g kg^−1^ (Fig. 2p).

### Shifts of seed composition

**Figure 3:**
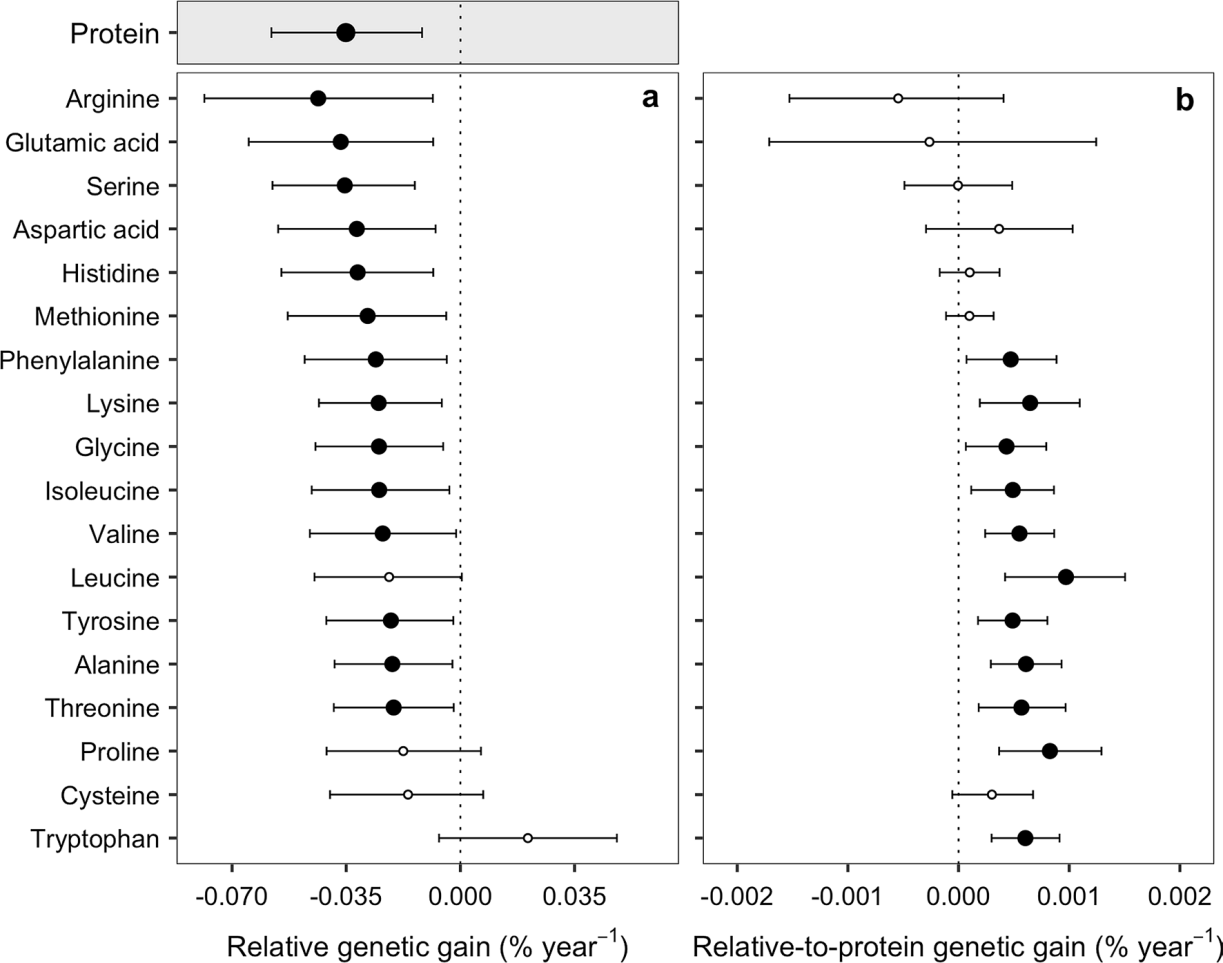
Relative genetic gain of protein and amino acid concentrations (**a**) and the relative-to-protein genetic gain of amino acid concentrations (**b**), calculated from 13 soybean genotypes released from 1980 to 2014 period. Points represent the medians of bootstrapped distribution with their respective 95% confidence intervals (small horizontal lines). Black solid points represent the AAs with significant relative gains, whereas the empty points are non-significant (95% CI including zero).

In order to compare the different slope magnitudes between protein and all AAs, each individual slope of absolute genetic gain was divided by the last fitted value (Fig. 3), thereby resulting in a relative gain. The estimated relative genetic gain of protein was − 0.035% year^−1^ (Fig. 3a). The confidence intervals of leucine, proline, cysteine and tryptophan overlapped the 0% genetic gain threshold, and therefore, were considered non-significant (empty points). The remaining AAs all decreased in concentration over time (black solid points). The magnitude of gain is provided here from the largest negative gain to the smallest in the following order: arginine (− 0.044%), glutamic acid (− 0.037%), serine (− 0.035%), aspartic acid (− 0.032%), histidine (− 0.032%), methionine (− 0.028%), phenylalanine (− 0.026%), lysine (− 0.025%), glycine (− 0.025%), isoleucine (− 0.025%), valine (− 0.024%), tyrosine (− 0.021%), alanine (− 0.021%), and threonine (− 0.020%) (Fig. 3a).

Not all AAs presented a negative trend of the same magnitude of seed protein as portrayed by the relative-to-protein genetic gain (Fig. 3b). This relationship is presented by the individual AA relative concentration to protein (%) and genotype year of release. Some AAs presented a less than proportionate reduction relative-to-protein and thus, those AAs were clustered as less negatively affected by the overall decrease in protein (black solid points). Within this cluster, we found the following AAs: phenylalanine, lysine, glycine, isoleucine, valine, leucine, tyrosine, alanine, threonine, proline, and tryptophan. In a second cluster, we found AAs presenting a decreasing relative-to-protein concentration but not significantly different from zero. They are arginine, glutamic acid, serine, aspartic acid, histidine, methionine, and cysteine (empty points) (Fig. 3b).

### Relationship between protein and amino acid concentration in historical genotypes

**Figure 4:**
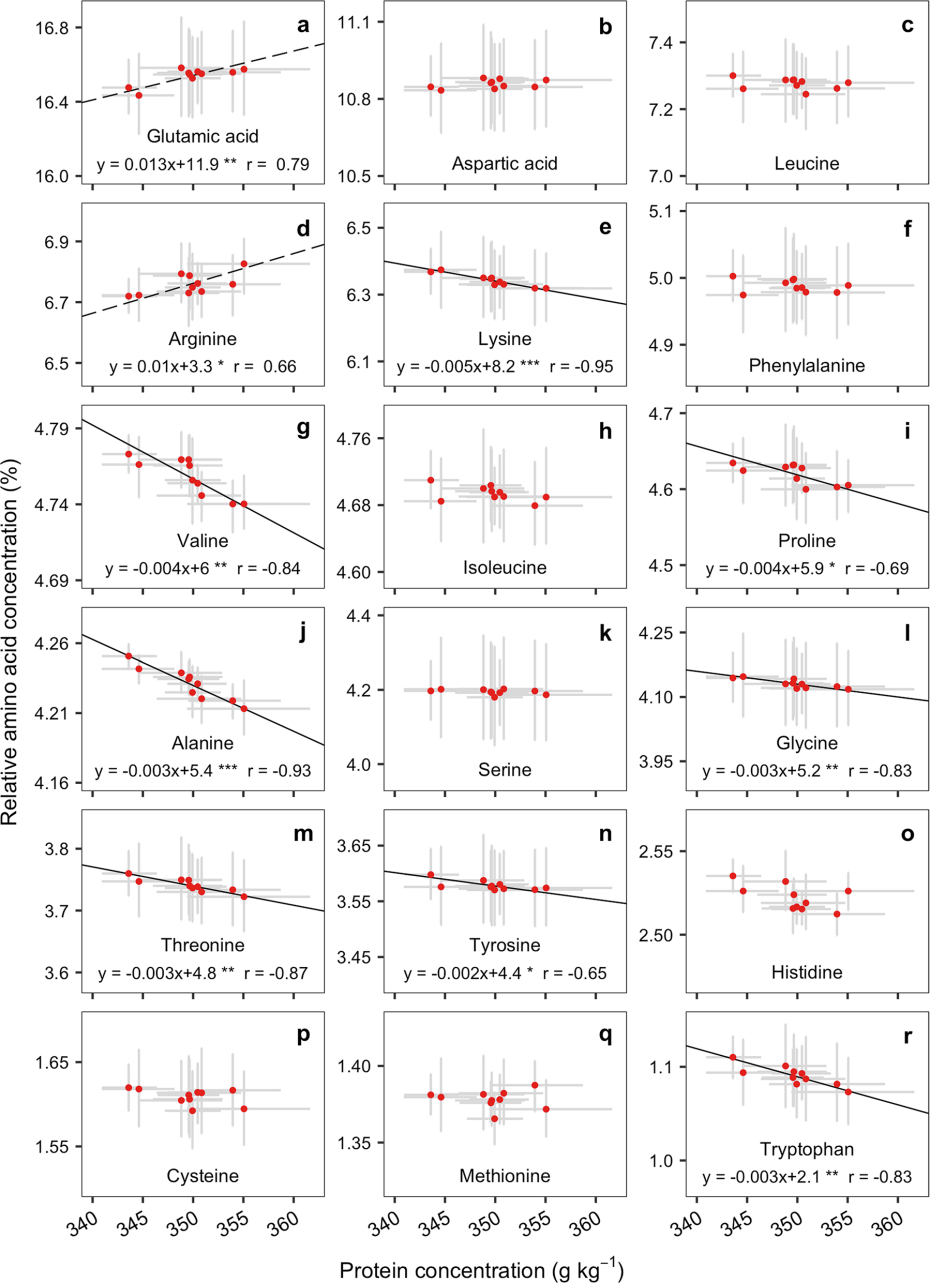
Relationship between glutamic acid (**a**), aspartic acid (**b**), leucine (**c**), arginine (**d**), lysine (**e**), phenylalanine (**f**), valine (**g**), isoleucine (**h**), proline (**i**), alanine (**j**), serine (**k**), glycine (**l**), threonine (**m**), tyrosine (**n**), histidine (**o**), cysteine (**p**), methionine (**q**), tryptophan (**r**), and protein concentration in seed of genotypes released from 1980 to 2014. Each point represents the average amino acid concentration between the two N treatments and genotypes (if more than one per year). Solid black line indicates the fitted model when the slope is negative, whereas dashed line indicates models with positive slope (*P* < 0.05)**.** Asterisks indicate significance of the coefficient: ***P < 0.001; **P < 0.01; *P < 0.05. *The* r *value* represents the Pearson correlation*.*

The estimated correlation between an AAs relative concentration (%) and protein concentration (g kg^−1^) was significant (*P* < 0.05) in 10 out of 18 AA in seeds from historical soybeans genotypes (Fig. 4). Glutamic acid and arginine were the only AAs to show a positive slope (Fig. 4a, d). The rate of increase was 0.013 and 0.010% for glutamic acid and arginine, respectively. The remaining 8 AAs with a significant relationship to protein concentration demonstrated a negative slope. This was the case for lysine (− 0.005%), valine (− 0.004%), proline (− 0.004%), alanine (− 0.003%), glycine (− 0.003%), threonine (− 0.003%), tyrosine (− 0.002%), and tryptophan (− 0.003%). Aspartic acid, leucine, phenylalanine, isoleucine, serine, histidine, cysteine, and methionine had no significant relationship to protein concentration in the historical genotypes.

## Discussion

Our results highlight the historical trends (1980–2014) in seed AAs concentrations. Yield improvement and protein reduction were within the range reported in the literature^5,21–25^. In addition, as previously documented for soybeans in maturity group III^20^, the rate of protein reduction over years was unchanged by the application of N fertilizer (670 kg ha^−1^). Fourteen of the 18 AAs analyzed were present in lower concentrations in more recently released genotypes (Figs. 2, 3a). The shifts of the most abundant AAs in soybean (glutamic acid and aspartic acid) were in the same range as the protein reduction rate (Fig. 3a). Alternatively, the concentrations of the essential AAs lysine and threonine increased relative to protein which may represent an impact on the nutritional quality of soybean meal^4^. Although a majority of the AAs decreased in absolute values, 11 AAs increased in concentrations relative-to-protein (Fig. 3b), including leucine, isoleucine, histidine, phenylalanine, and valine, which are essential AAs for animal nutrition^12,26^. Therefore, breeding efforts to develop high protein genotypes should consider the underlying impact on AAs and the potential impact on the nutritional value of the seeds^6,27^.

Addition of N did not affect protein or AA shifts over time (Table 1). A significant effect of N fertilization on protein is more likely in environments with poor N supply such as greenhouse conditions^16^ or low activity of biological nitrogen fixation^28^. In field studies, N biological fixation resulting from seed inoculation or indigenous soil rhizobia infection, may provide sufficient N to support high crop performance^29–31^. For the tested yield levels, our results indicate the inability of N fertilization to reverse the decline on soybean seed protein and AAs concentrations. A similar outcome was presented by Wilson et al. (2013), documenting a protein decline of − 0.25 g kg^−1^ year^−1^ for soybean genotypes released between 1923 and 2008 under contrasting N rates (zero vs. 560 kg N ha^−1^). Regarding the AA profile, controlled condition studies support a positive relationship between supra-optimal N and essential^32^ or storage AAs^33^. For non-leguminous crops, field studies have validated the concept of N application enhancing AAs concentrations^34–36^. For soybeans, however, only a few studies were carried out in the field with N fertilization, and the results showed an increase in AAs concentrations only under low N availability^28^ or associated with sulfur AAs reduction^37^. These findings were not observed in our results, therefore highlighting a lack of effect of non-limiting N supply for offsetting protein and AAs depression in historical soybean genotypes.

Additionally, protein concentration was shown to be a better predictor of changes in AAs over time, but only for a select few such as glutamic acid, arginine, lysine, valine, proline, alanine, glycine, threonine, tyrosine, and tryptophan (Fig. 4). Using protein as a predictor of the AA changes over time was previously reported^9^, but considering only modern genotypes rather than a historical set as presented in this current study. Our findings show similar relationships for some AA changes such as glutamic acid and arginine relative-to-protein genetic gain (Fig. 3b), but other AAs did not exactly follow the trend of protein, e.g., aspartic acid, leucine, phenylalanine, isoleucine, serine, histidine, cysteine, and, methionine (Fig. 4). To date, there are no reported predictive models describing the entire AA profile as a function of protein concentration (as a reference seed composition fraction). Therefore, establishing foundational prediction models for AAs will assist breeders and ultimately growers in delivering soybean genotypes focusing on specific market demands.

## Conclusions

This research explored the shifts in protein and AAs due to the genetic improvement of soybean genotypes from 1980 to 2014. These shifts were not driven by an increased N supply via inorganic N, as the N fertilization treatment did not change any trends for AAs concentrations. Similar negative rates, in absolute concentrations, were observed for some AAs such as arginine and glutamic acids but not for the rest of the AA profile relative-to-protein. Therefore, the concept of utilizing seed protein concentration genetic gain as an indicator of potential changes in AAs is not a valid rationale. Emerging areas of research focusing the genetic control of amino acids synthesis and its interaction to the environment will provide the foundation for improving seed traits either maintaining or improving the nutritional value of soybean.

## Methods

### Field conditions and experimental design

Two field experiments were conducted at the Kansas River Valley research station in Rossville, Kansas, United States (39°07´ N; 95°55´ W) during the 2016 and 2017 growing seasons. The local weather is Dfa continental humid with hot and wet summers^38^. Temperature in the 2016 and 2017 seasons averaged 22 and 23 °C, respectively. The seasonal precipitation was 450 mm in 2016 and 523 mm in 2017. The experimental area was kept primarily under rainfed conditions, although 345 mm and 221 mm (2016 and 2017, respectively) of water was supplemented as needed to avoid potential drought stress. The soil type was a Fluventic Hapludoll with the following chemical attributes at a 0–0.15 m depth: pH 6.9 (2016) and 7.3 (2017); organic matter (%): 2.2 (2016) and 1.3 (2017); N-nitrate (mg kg-^1^): 3.0 (2016) and 2.7 (2017); Cation exchange capacity (cmol_c_ dm^−3^): 11 (2016) and 5.8 (2017); phosphorous Mehlich (mg kg^−1^): 21 (2016) and 13 (2017); potassium (mg kg^−1^): 153 (2016) and 90 (2017); calcium (mg kg^−1^): 2074 (2016) and 951 (2017); magnesium (mg kg^−1^): 202 (2016) and 95 (2017). The area is permanently maintained under a maize-soybean rotation.

The experimental design was a randomized complete block in split-plot arrangement with four replications in both seasons. The main-plot consisted of the N factor with two levels and the sub-plot was the genotype factor with 13 levels. The N treatments were N fertilization at a rate of 670 kg N ha^−1^ and the control without N (0 N). The N fertilizer was equally split at sowing, R1, and R3 phenological stages^39^ as a side dressed application of liquid urea ammonium nitrate (N P K, 28-0-0). Seed inoculation was performed shortly before sowing with the application of 3 × 10^9^ colony units of *Bradyrhizobium japonicum* per 1 kg of seeds.

The subplot consisted of 13 genotypes released between 1980 and 2014 with maturity groups (MG) ranging between 3.0 and 4.0. Genotypes, associated MG and release dates are as follow: P3981 (1980—MG 3.0), Williams 82 (1981—MG 3.0), 9391 (1987—MG 4.0), 9392 (1991—MG 3.8), P93B82 (1997—MG 3.8), 93B67 (2001—MG 3.9), 93M90 (2003—MG 3.0), P93Y92 (2009—MG 3.9), 94Y23 (2013—MG 4.0), P35T58R (2013—MG 3.0), P39T67R (2013—MG 4.0), P31T11R (2014—MG 3.1), and P34T43R (2014—MG 3.4). Planting dates were May 12 in 2016 and May 18 in 2017. The plot size was 10 m long by four 0.76 m rows. The experimental area was kept free of weeds, pests, and diseases.

### Seed yield, protein and amino acids determination

At harvest maturity (R8), the two center rows in each plot were harvested with a plot combine, and the seed yield was adjusted to 130 g kg^−1^ water content basis. Approximately one kilogram of seed was sampled from each plot to measure seed composition. After seeds were dried to constant weight, the samples were ground to 0.1 mm final particle size. Protein and AAs concentrations were estimated with near-infrared spectroscopy (NIR) using the Perten DA7200 Feed Analyzer (Perten Instruments, Stockholm, Sweden). Briefly, the raw ground material was scanned between 1000 and 2500 nm wavelength and the reflectance normalized to a reference ceramic plate. The readings were subject to error removal due to an eventually uneven cup filling or sample size heterogeneity. The calibration between normalized reflectance and AA concentration was cross-validated using standard samples analyzed by wet chromatography following the AOAC 982.30 method^40^. The calibration curves were tested by root-mean-square error (RMSE). This method estimates the protein and 18 AAs concentrations (g kg^−1^) corrected to water content. However, this method does not distinguish between asparagine and aspartate, or between glutamine and glutamate. Therefore, glutamic acid and aspartic acid forms were reported as the sum of their respective components.

The absolute genetic gain (g kg^−1^ year^−1^) was estimated by the regression of yield, protein, or each individual AA to genotype year of release. The relative genetic gain (% year^−1^) was calculated to allow the comparison between amino acids and protein concentrations. Thus, the slope of the absolute genetic gain for each AA was divided by the most recent estimated concentration^41^ (Eq. 1).

1 $$\mathrm{Relative\ genetic\ gain}\left({\%}\,{\mathrm{year}}^{-1}\right)= 100\times \left(\frac{\mathrm{Absolute\ genetic\ gain} \left(\mathrm{g\ kg}^{-1} \,{\mathrm{year}}^{-1}\right)}{\mathrm{Most\ recent\ estimated\ concentration} \,\left(\mathrm{g\ kg}^{-1}\right)}\right)$$

The relative-to-protein genetic gain (% year^−1^) was determined by the relationship between the relative to protein concentration ratio (Eq. 2) with genotype year of release.

2 $$\mathrm{Relative\ protein\ ratio}\,\left({\%}\right)=100\times \frac{\mathrm{Seed\ AA\ concentration}\, \left(\mathrm{g\ kg}^{-1}\right)}{\mathrm{Protein\ AA\ concentration }\left(\mathrm{g\ kg}^{-1}\right)}$$

Finally, to investigate the correlation between AAs and protein regardless the year of release, AA concentrations relative-to-protein (Eq. 2) were tested against protein concentration.

### Data analysis

We first tested the effect of N treatment by fitting two linear mixed models for each variable. The first model included the year of release, N treatment, and the interaction as fixed effect factors, and the second model included only the year of release as the fixed effect. As N treatment was not significant, the model with the lowest score for Akaike Information Criterion (AIC) was selected. The random factors included year, block nested in year, N treatment nested in the interaction of block with year, and genotype nested in the interaction of N treatment, block, and year. Models were fitted using the package “lme4”^42^ within the R software^43^. Assumptions of normality and homogeneity of the residuals were checked and no transformation was required.

The Resampling with Replacement Bootstrap was used to estimate the slope coefficient and the empirical distribution of model estimators^44^. A total of 5000 iterations were performed. All the distributions were summarized by the median, and the 2.5 and 97.5% percentiles were used as the boundaries of the 95% confidence intervals (CI), allowing statistical inference on the parameters^45^. The Pearson correlation coefficient (r) was estimated from the variable median estimation for each year of release (means distribution). The relative-to-protein genetic gain and relative genetic gain were empirically clustered using confidence intervals different from zero to separate increasing, neutral, or decreasing trends over the years. The standardized major axis (sma) regression was fitted^46^ to test the relationship between AAs concentrations relative to protein and protein concentration. Data visualization (Figs. 1, 2, 3, 4) was performed using the package “ggplot2”^47^ within the R software^43^.
